# Incidence and Functional Outcomes of Scapholunate Diastases Associated Distal Radius Fractures: A 2-year Follow-Up Scapholunate Dissociation

**DOI:** 10.2174/1874325001812010033

**Published:** 2018-01-31

**Authors:** Jonathan Lans, Alejandro Lasa, Neal C. Chen, Jesse B. Jupiter

**Affiliations:** 1 Department of Orthopedic Surgery, Hand and Upper Extremity Service, Massachusetts General Hospital, Harvard Medical School, Yawkey Center, Suite 2100, 55 Fruit Street, Boston, MA, 02114, USA; 2 Department of Traumatology, British Hospital, Avenida Italia 2420, 11600 Montevideo, Uruguay; 3 Department of Orthopedic Surgery, Hand and Upper Extremity Service, Massachusetts General Hospital, Harvard Medical School, Yawkey Center, Suite 2100, 55 Fruit Street, Boston, MA, 02114, USA

**Keywords:** Scapholunate ligament, Scapholunate dissociation, Distal radius, SLAC, Wrist osteoarthritis, Patient reported outcomes

## Abstract

**Background:**

The Scapholunate Interosseous Ligament (SLIL) is the first intrinsic carpal ligament to be injured in wrist trauma, present in up to 64% of the distal radius fractures. However, it remains unclear what patients develop symptoms, making primary treatment of these injuries accompanying distal radius fractures remains questionable.

**Objective:**

The aim of this study was to evaluate the functional outcomes of patients with scapholunate diastasis associated with distal radius fractures.

**Methods:**

We evaluated 391 patients with a distal radius fracture. Using Computer Tomography (CT) scans the scapholunate interval was measured. We identified 14 patients with an SLD (>3mm) of the injured wrist, which underwent a CT-scan of the contralateral wrist. To evaluate the functional outcomes at a mean follow up of 136±90 weeks, we used the Quick Disabilities of the Arm, Shoulder and Hand (qDASH) Score.

**Results:**

There were 8 patients with bilateral SLD and 6 patients with unilateral SLD. Five patients had a qDASH score of 0 and one patient showed a qDASH score of 18.2. The patient with a poor score had bilateral preexisting osteoarthritis of the wrist. No patient had additional surgery of the SLIL.

**Conclusion:**

In patients with distal radius fractures, more than half of the 14 patients with an SL gap on CT had widening on the contralateral side. It is therefore worthwhile to image the contralateral wrist before diagnosing a SLD. The patients with unilateral SLD should not be surgically treated at initial presentation because they may have good functional outcomes after a follow up of 2 years.

## 1 INTRODUCTION

Scapholunate Interosseous Ligament (SLIL) injury accompanies intra-articular radius fractures in 5-64% [1-8]. It is an important stabilizer of the carpal motion between the scaphoid and lunate [9]. Injury of the SLIL can lead to scapholunate dissociation (SLD) eventually leading to a scapholunate advanced collapse (SLAC) of the wrist in some cases [6, 10, 11]. These changes in wrist biomechanics can lead to carpal osteoarthritis along a predictive path [12-14]. It is therefore important to perform scapholunate ligament reconstruction before this cascade begins. However, it is unpredictable what patients will develop symptoms [15].

In the acute posttraumatic radiographs of distal radius fractures, a scapholunate diastasis (>3mm) is often seen [8, 16, 17]. However, these are not always clinically relevant. Possibly, ligamentous healing takes place during the immobilization [4, 18, 19]. Nonetheless, some patients receive simultaneous open distal radius fixation and direct SLIL reconstruction [20-24].

Through our clinical experience, we believe that few of the patients with acute scapholunate diastasis actually develop symptoms. The aim of this study was to determine the incidence of SLD’s associated with distal radius fractures and to evaluate the functional outcomes of these patients.

## 2 MATERIALS AND METHODS

### 2.1 Study Population

After ethics committee approval, we retrospectively evaluated all distal radius fractures (n=391) from 2007 to 2015 that were treated in the British Hospital of Montevideo, Uruguay. These were identified through an ongoing procedure database kept by the traumatology department, that collects patient and treatment data prospectively. Patients with a non-anatomic fracture position were operatively treated using a volar approach with volar variable angle plate. The scapholunate joint was not surgically treated in any patient. All patients underwent a postoperative Computer Tomography (CT) scan. We excluded one patient with a bilateral distal radius fracture that had a SLD of one of the fractured sides. Patients (n=14) with a scapholunate interval (SLI) of ≥3mm in the injured wrist underwent a CT scan of the contralateral wrist. Of these patients 8 (57%) had a contralateral SLI that was ≥3mm. We included 2 males and 4 females, with a median age of 61 (54-87) years with an average radiographic follow-up of 109±91 weeks and average functional follow-up of 136±90 weeks. One patient had a conventional radiograph at final follow-up, all other patients had a CT scan. There were 3 type C3 fractures, 1 type C1 fracture, 1 type B2 fracture and 1 type A2 fracture. There was an associated ulna fracture in 2 patients.

### 2.2 Evaluation

**Fig. (1):**
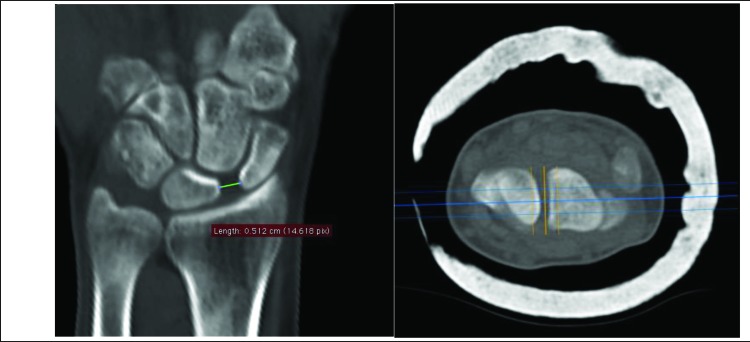
Measurement of the scapholunate interval in the coronal plane

**Fig. (2):**
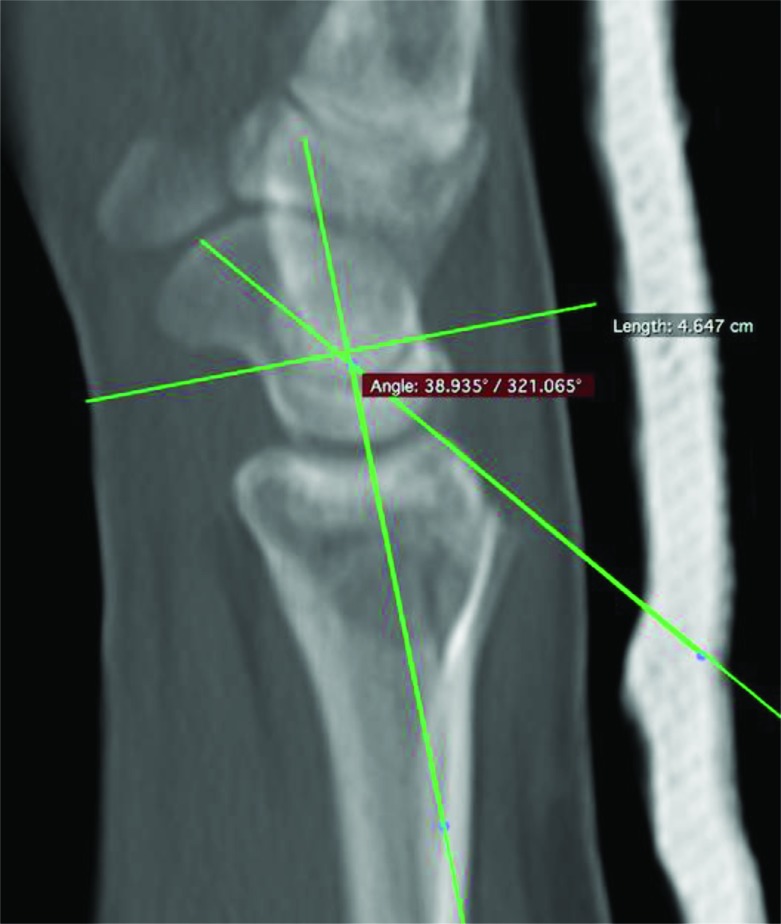
Measurement of the scapholunate angle in the sagittal plane.

Computer tomography scan (SOMATOM Emotion 16, Siemens Healthcare, Malvern, Pennsylvania, USA) images of the wrist were obtained directly postoperatively and at final radiographic follow-up. Measurements were performed using OsiriX Viewer (Pixmeo SARL, Bernex, Switzerland). The scapholunate interval was measured positioning the CT beam parallel to the SLI in the axial plane (Fig. **1**). The CT slices were digitally increased to a width between 7-14mm to evaluate the SLI and the scapholunate angle (SLA) in the mean modus. We measured the SLI from the midpoint of the lunate to the midpoint of the scaphoid in the coronal plane. The SLA was evaluated in a sagittal plane by calculating the angle between the scaphoid axis and the lunate axis (Fig. **2**). The functional outcome was evaluated using the Quick Disabilities of the Arm, Shoulder and Hand (qDASH) was used.

### 2.3 Statistical Analysis

Continuous data were stated as mean ± standard deviations and categorical data as frequencies and percentages. We used a linear regression to compare the differences between SLI distances and SLA in the postoperative CT scan compared to the CT scan at final follow-up.

## 3 RESULTS

The average postoperative SLI was 4.1±0.4mm and the average SLI at final follow-up it was 4.3±0.9mm (*p=*0.64). The average postoperative SLA was 63.8±5.0° and at final follow-up this as 63.6±6.9° (*p=*0.33). Five patients had a Quick DASH score if 0 and one patient had a score of 18.2, this patient had severe preexisting degenerative osteoarthritis of the injured wrist. No patient required additional surgery of the SLIL.

## 4 DISCUSSION

In this study, we evaluated the scapholunate interval (SLI) in 391 patients with a distal radius fracture that underwent volar plate fixation. Using a computer tomography of the wrist we measured the SLI defining a scapholunate diastasis when the SLI was ≥3mm. Of all the distal radius fractures, there were 14 patients with a SLD, of which 8 showed a SLI of ≥3mm in the contralateral wrist. We identified 6 patients with unilateral SLD’s, possibly due to a SLIL tear. This highlights the importance of bilateral imaging as is the case in other uncommon traumatic conditions of the wrist [25-27]. None of the patients with an SLD had treatment of their scapholunate joint. After an average follow-up of more than 2 years months 5/6 patients reported an excellent DASH score. The patient with poor functional outcome had preexisting bilateral degenerative osteoarthritis of both wrists, experiencing more pain in the uninjured wrist (Tables **1** and **2**).

However, we need to interpret these results in respect to its strengths and limitations. First, we did not compare the outcomes of patients with an SLD to those without an SLD. Second, the measurements were performed by one author. Thirdly, CT scans are not standard clinical practice in all settings and our results are not applicable for conventional radiographs. The advantage of using CT scans is that it allows the evaluator to precisely position the radiographic beam parallel to the scapholunate joint avoiding measurement errors due to angulated views of the wrist. Suzuki *et al*. reported a sensitivity of 75% and specificity of 90% using computer tomography scans to measure the SLI [28]. The value of this study is that this is the first study using bilateral CT scans to compare the SLI after a distal radius fracture. Additionally, gives insight in the subjective outcomes of untreated SLD in patients with a distal radius.

Distal radius fractures are often accompanied with ligamentous injury, most frequently triangular fibrocartilage complex injuries followed by the SLIL injuries [1, 3, 5, 22, 29-35]. The scapholunate interosseous ligament is the most important stabilizer of the scapholunate joint and is the first to tear when axial loading of the wrist occurs [9, 36]. Mudgal and Jones emphasized to be aware for a SLIL injury in the presence of a fracture line between the scaphoid- and lunate fossa [23]. Furthermore, Mudgal advised treatment of scapholunate diastases when present as part of a four-part distal radius fractures [24]. Others have suggest scapholunate fixation for Geissler grade 3 and grade 4 SLIL tears [20-22]. It is however, still unknown what patients with SLIL injury develop scapholunate advanced collapse (SLAC) and what patients develop symptoms [6, 10]. In a study by Fassler *et al*. 73% of the patients with radiographic evidence for a SLAC did not report pain [11].

The golden standard to diagnose scapholunate tears is by arthroscopy of the wrist [37]. Using this modality, scapholunate tears associated with distal radius fractures occur in 7-64% patients [1-3, 5, 6, 20-22, 29, 32, 33, 35, 38-41]. The incidence of concomitant SLIL is lower in extra-articular fractures (7-33%) [2-4, 42]. Arthroscopically assisted distal radius fracture treatment allows confirmation of SLIL tears, but many of these lesion seem to be clinically irrelevant [1, 6]. Repeated arthroscopies to evaluate ligament healing would be ideal but are an unacceptable burden for patients and have therefore not been performed. In conventional anteroposterior radiographs SLD is often defined as a SLI of equal to or more than 2- or 3mm. Incidences reported using ≥2mm range 26-52% whereas studies using ≥3mm report incidences of 5-8%. After distal radius fracture reduction many SLD’s may resolve, possibly due to ligamentotaxis [4].

Geissler *et al*. showed that Geissler grade 3 or 4 lesions had a radiographic SLI of ≥3mm [35]. Therefore we chose ≥3mm to define a SLD as we considered this clinically relevant. Using the CT scan we found an incidence of SLD in 1.5% of the patients, which is lowest incidence reported to date. Furthermore, we found that 57% of the SLD’s were bilateral which is similar as in literature, where this has been reported to be present in 38-52% [10, 43]. We believe that using a radiographic cut-off for SLD of ≥2mm diagnoses many clinically irrelevant increased scapholunate intervals.

Outcomes regarding SLD’s associated with distal radius fractures are contradicting. Several reports with a follow-up of 1-year have shown no differences between patients with or without SLD accompanying a distal radius fracture [1, 29, 42]. On the contrary Tang *et al*. showed significantly increased SLI’s after one year follow-up in 20 patients treated with cast immobilization and that 8 patients had symptoms requiring SLIL treatment after one year [17]. They suggested that a SLI of ≥3mm along with the presence of additional signs of carpal instability such as the cortical ring sign, a scapholunate angle >70° and foreshortening of the scaphoid are indicative for severe injury. In our eyes defining the precise origin of wrist pain in a posttraumatic wrist is difficult, especially attributing pain to the scapholunate joint. Further support for worse outcomes in patients SLIL tears has been reported by Kasapinova *et al*. but in this study the follow-up was only 6 months and it compared inhomogeneous treatment groups [21]. This makes it difficult to generalize their outcomes. In a series of 15 patients with type B fractures Yoshida *et al*. arthroscopically confirmed SL tears 14 patients and suggested that pinning Geissler grade 3 and 4 leads to better outcomes at a mean follow-up of 11.8 months [44]. It is however difficult to change practice based on these results because three patients that were pinned were compared to three that were not pinned, without any statistical analysis.

With regard to long-term follow up, the outcomes are in favor of conservative treatment of SLD’s associated with distal radius fractures. Mrkonjic *et al*. evaluated arthroscopically diagnosed scapholunate tears after 13-15 years of follow up after conservative treatment [6]. They concluded there was no difference in objective or subjective outcomes in patients with Geissler grade 3 tears compared to grade 1-, grade 2- or no tears. These findings were confirmed by Finsen *et al*. that defined SLD as a SLI ≥2mm in conventional radiographs of extra-articular distal radius fractures and evaluated the outcomes of 12 patients after a mean follow-up of 6 years [45]. They concluded that the outcomes with or without SLD were similar, although statistical analysis was lacking. Added to that, scapholunate fixation along with distal radius fixation does not seem to improve functional outcomes at 4 years follow up [46].

## CONCLUSION

In conclusion, we have highlighted the importance of bilateral wrist imaging as 57% of the wrists showed bilateral scapholunate intervals of ≥3mm. Furthermore, we showed that patients with distal radius fractures that underwent surgical volar plate fixation and no treatment of the scapholunate interosseous ligament had excellent patient reported outcomes. Distal radius fractures are not associated with a high incidence of clinically significant SLI and when one is detected the opposite wrist is likely to show a similar finding. Therefore, initial treatment of a SLI of ≥3mm does not always require treatment when accompanying a distal radius fracture. Further studies addressing long term functional outcomes are needed to increase our understanding of the consequences of scapholunate interosseous ligament injuries.

## Figures and Tables

**Table 1:** Patient characteristics with unilateral scapholunate dissociation.

**Variable**	**All Patients **
	**n=6**
**Age, median (IQR), years**	61 (54-87)
**Male sex, n(%)**	2 (33.3)
**Right, n(%)**	3 (50.0)
**AO Classification, n(%)**	
A2	1 (16.7)
B3	1 (16.7)
C1	1 (16.7)
C3	3 (50.0)
**Ulnar styloid fracture, n(%)**	2 (33.3)

**Table 2:** Outcomes.

**Case**	**Follow-up**	**SLD (mm)**		**SLA (°)**		**Quick DASH**
-	*(weeks)*	*Initial*	*Final*	*Initial*	*Final*	-
1	271	4.4	6.1	67.3	57.4	0
2	226	4.3	3.3	60.5	60.3	18.2
3	92	4.0	3.9	54	63.1	0
4	102	3.5	4.3	58.6	62.3	0
5	70	3.7	4.1	70.6	70.5	0
6	52	4.5	4.3	70.8	68.9	0
*p-value*	-	0.64		0.33		-

## LIST OF ABBREVIATIONS

CT: Computer Tomography
qDASH: Quick Disabilities of the Arm, Shoulder and Hand
SLAC: Scapholunate Advanced Collapse
SLD: Scapholunate Dissociation
SLI: Scapholunate Interval
SLIL: Capholunate Interosseous Ligament
